# *Lactobacillus plantarum* PS128 prevents cognitive dysfunction in Alzheimer’s disease mice by modulating propionic acid levels, glycogen synthase kinase 3 beta activity, and gliosis

**DOI:** 10.1186/s12906-021-03426-8

**Published:** 2021-10-09

**Authors:** Hei-Jen Huang, Jie-Ling Chen, Jian-Fu Liao, Yu-Hsin Chen, Min-Wei Chieu, Ya-Yun Ke, Chih-Chieh Hsu, Ying-Chieh Tsai, Hsiu Mei Hsieh-Li

**Affiliations:** 1 Department of Nursing, MacKay Junior College of Medicine, Nursing and Management, Taipei, 11260 Taiwan; 2 Department of Life Science, National Taiwan Normal University, Taipei, 11677 Taiwan; 3 Institute of Biochemistry and Molecular Biology, National Yang Ming Chiao Tung University, Taipei, 11221 Taiwan; 4 Bened Biomedical Co., Ltd., Taipei, 10448 Taiwan

**Keywords:** PS128 psychobiotic, Streptozotocin, Propionic acid, Gliosis, Glycogen synthase kinase 3 beta, Cognition

## Abstract

**Background:**

According to recent evidence, psychobiotics exert beneficial effects on central nervous system-related diseases, such as mental disorders. *Lactobacillus plantarum* PS128 (PS128), a novel psychobiotic strain, improves motor function, depression, and anxiety behaviors. However, the psychobiotic effects and mechanisms of PS128 in Alzheimer’s disease (AD) remain to be explored.

**Objectives:**

The goal of the current study was to evaluate the beneficial effects of PS128 and to further elucidate its mechanism in AD mice.

**Methods:**

PS128 (10^10^ colony-forming unit (CFU)/ml) was administered via oral gavage (o.g.) to 6-month-old male wild-type B6 and 3 × Tg-AD mice (harboring the PS1M146V, APPswe and TauP30IL transgenes) that received an intracerebroventricular injection of streptozotocin (icv-STZ, 3 mg/kg) or vehicle (saline) for 33 days. After serial behavioral tests, fecal short-chain fatty acid levels and AD-related pathology were assessed in these mice.

**Results:**

Our findings show that intracerebroventricular injection of streptozotocin accelerated cognitive dysfunction associated with increasing levels of glycogen synthase kinase 3 beta (GSK3β) activity, tau protein phosphorylation at the T231 site (pT231), amyloid-β (Aβ) deposition, amyloid-β protein precursor (AβPP), β-site AβPP-cleaving enzyme (BACE1), gliosis, fecal propionic acid (PPA) levels and cognition-related neuronal loss and decreasing postsynaptic density protein 95 (PSD95) levels in 3 × Tg-AD mice. PS128 supplementation effectively prevented the damage induced by intracerebroventricular injection of streptozotocin in 3 × Tg-AD mice.

**Conclusions:**

Based on the experimental results, intracerebroventricular injection of streptozotocin accelerates the progression of AD in the 3 × Tg-AD mice, primarily by increasing the levels of gliosis, which were mediated by the propionic acid and glycogen synthase kinase 3 beta pathways. PS128 supplementation prevents damage induced by intracerebroventricular injection of streptozotocin by regulating the propionic acid levels, glycogen synthase kinase 3 beta activity, and gliosis in 3 × Tg-AD mice. Therefore, we suggest that PS128 supplementation is a potential strategy to prevent and/or delay the progression of AD.

**Supplementary Information:**

The online version contains supplementary material available at 10.1186/s12906-021-03426-8.

## Background

Alzheimer’s disease (AD) is characterized by progressive and irreversible memory loss that results in a substantial burden on the patient and caregivers. The pathological features of AD include the progressive accumulation of parenchymal senile plaques consisting of amyloid-β (Aβ) protein and intracellular neurofibrillary tangles (NFTs) composed of abnormal phosphorylated tau, which induce chronic gliosis primarily in cognitive brain areas, and subsequently lead to synaptic damage, cognitive impairment, and neurodegeneration [1–3]. However, the multifactorial nature of AD makes the treatment of AD challenging [4]. To date, anti-amyloid and anti-tau agents have not been successful in clearing or preventing AD pathology [5]. Therefore, an urgent need is to develop viable therapeutics and preventive treatments to improve the quality of life of patients with AD and to decrease the burden of caregivers.

The decreased diversity of the gut microbiota in patients with AD has attracted interest in recent years [6–8]. The term “microbiota-gut-brain axis” has also been used to describe the association between gut microbiota and AD through various routes, including the immune system, tryptophan metabolism, microbial metabolites, the vagus nerve and the enteric nervous system [7]. Evidence suggests that short-chain fatty acids (SCFAs), including butyric acid (C4), propionic acid (C3), acetic acid (C2), and microbial metabolites in the colon, attenuate the neuropathological features of AD and other neurodegenerative diseases by providing an alternative energy source to rectify brain hypometabolism [9]. Additionally, selected SCFAs might modulate neuroinflammation, which is a major pathomechanism of the early and preclinical course of AD [10, 11]. Based on accumulating evidence, long-term dietary supplementation with probiotics containing multiple strains improves cognitive dysfunction in individuals with AD [12, 13]. *Lactobacillus plantarum* MTCC1325 and *Lactobacillus pentosus var. plantarum* (C29) were also shown to attenuate memory impairment in D-galactose-induced AD animal models [14, 15]. The reports described above suggest that probiotic supplementation may be an effective method to delay AD progression. *L. plantarum* PS128 (PS128) is a psychobiotic isolated from fu-tsai, a traditional Taiwanese fermented vegetable food product that has been reported to reduce anxiety and depression by decreasing inflammation and cortisol levels [16, 17]. Recent evidence also suggests that PS128 psychobiotics attenuate hyperactive behaviors by modulating the microbiota-gut-brain axis [18]. However, the relationship between SCFAs derived from microbial metabolites and PS128 psychobiotics in individuals with AD remains unclear.

An intricate relationship exists between the gut microbiota and the development of types 1, 2 and 3 diabetes [19]. Type 3 diabetes has been proposed as a common term for AD [20]. The impairment of brain glucose uptake/metabolism is a common early abnormality in individuals with AD [21]. Accumulating evidence also reveals an important role for brain glucose hypometabolism in the process of cognitive decline and aging [22–25]. An intracerebroventricular injection of the diabetogenic toxin streptozotocin (icv-STZ) exacerbates memory disturbances and AD-like neurochemical changes in the brains of 3 × Tg-AD mice (harboring the PS1M146V, APPswe and TauP30IL transgenes) by impairing insulin signaling [26, 27]. Therefore, icv-STZ was used to accelerate AD progression in 6-month-old male 3 × Tg-AD mice (the age before typical AD pathologies develop) in the present study. We aim to examine the beneficial effects of PS128 on 3 × Tg-AD mice treated with icv-STZ and to elucidate the role of SCFAs in 3 × Tg-AD mice treated with PS128 and icv-STZ. Our results showed that the administration of icv-STZ accelerated the disease progression of AD, which might result from gliosis occurring through increased GSK3β activity and fecal PPA levels in 3 × Tg-AD mice. PS128 supplementation effectively prevented the damage induced by icv-STZ by reducing the fecal PPA levels, GSK3β activity, and gliosis in 3 × Tg-AD mice.

## Methods

### Animals

C57BL/6 J male mice and 3 × Tg-AD mice on a B6/129 hybrid genetic background were purchased from the National Breeding Center for Laboratory Animals and Jackson Laboratory (004807; Bar Harbor, ME, USA). 3 × Tg-AD mice were backcrossed for more than 10 generations to the B6 background. Based on our previous results, we did not detect an obvious cognitive impairment or other typical pathological features in the 3 × Tg-AD male mice at 6 to 7 months of age [28]. Therefore, bilateral icv-STZ was used to exacerbate the progression of AD, as described in previous studies [26, 27]. The mice were housed at 20–25 °C with 50–60% relative humidity on a 12 h light/dark cycle, and food and water were available ad libitum. The body weight of mice was approximately 26–27 g. All experiments were performed during the light phase between 7:00 a.m. and 7:00 p.m. To minimize animal suffering, the mice were deeply anesthetized with avertin (0.4 g/kg body weight, Sigma; St. Louis, MO, USA) and then sacrificed by perfusion or cervical dislocation for pathological analyses after a series of behavioral tasks. All experimental procedures were approved by an ethics committee at the Institutional Animal Care and Use Committee (IACUC) of National Taiwan Normal University (Permit Number: 106036).

### Experimental design

The experimental timeline is shown in Fig. S1A. Wild-type B6 and 3 × Tg-AD mice aged 6 months were randomly divided into 6 groups (15–20 mice/group) according to the sigmoid distribution of body weight: (i) B6 mice treated with saline (oral gavage, o.g.)/saline (icv) as control group; (ii) B6 mice treated with PS128 (o.g.)/saline (icv); (iii) 3 × Tg-AD mice treated with saline (o.g.)/saline (icv); (iv) 3 × Tg-AD mice treated with PS128 (o.g.)/saline (icv); (v) 3 × Tg-AD mice treated with saline (o.g.)/STZ (icv); and (vi) 3 × Tg-AD mice treated with PS128 (o.g.)/STZ (icv). After adaption to handling and o.g. for 7 days (days 8–14), the mice were administered 100 μL of PS128 (o.g., daily, 10^10^ CFU/ml; Bened Biomedical; Taipei, Taiwan) or the saline vehicle for 33 days (days 8–41). The concentration of PS128 was selected according to previous studies [16, 17]. Seven days after PS128 administration, the mice were anesthetized with avertin (0.4 g/kg of body weight; Sigma) and received a single icv injection of 2 μL of STZ (3 mg/kg; Sigma) or vehicle (saline) into the bilateral ventricle (0.3 mm with respect to the bregma, 1.0 mm right and left of the central suture, 2.5 mm deep). Serial behavioral tests, namely, the open field test (OFT), elevated plus maze (EPM), Y maze and Morris water maze (MWM), were conducted on days 22–23, 26–27, 28–29, and 34–40, respectively. Finally, the mice were sacrificed for western blot, immunohistochemical, and SCFA analyses of fecal boli on day 41. Before sacrifice, mouse blood samples were obtained by a tail prick to measure glucose levels using a commercial glucometer (Accu-CheckActive; Roche, Mannheim, Germany).

### Behavioral assessments

Mice were subjected to a series of behavioral tasks (*n* = 15–20/group). All mice were habituated in the behavioral room for 30 min before testing, and all behavioral apparatuses were carefully cleaned between subjects with 70 and 30% ethanol sequentially to remove any olfactory cues. Behavioral data were recorded using a video camera and analyzed using an automatic tracking system (EthoVision-XT; Noldus, Wageningen, The Netherlands).

### Open field test (OFT)

The OFT was conducted using a recently described method [29]. Briefly, mice were individually placed in the central zone (15 × 15 cm) of a white acrylic box (30 cm long × 30 cm wide × 30 cm high) and then recorded for 10 min. The total distance travelled by the mice was measured as an index of exploratory activity, and a decrease in the time spent in the center of the open field arena was considered an index of anxiety-like phenotypes.

### Elevated plus maze (EPM)

The EPM was carried out as described in a recent study [29]. The EPM apparatus consisted of a plus-shaped maze constructed of white opaque acrylic with two opposing open arms (30 cm long × 5 cm wide) and two opposing closed arms (30 cm long × 5 cm wide × 15 cm high) connected by a central platform (10 cm long × 10 cm wide) and elevated 50 cm above the floor. The mouse was placed in the central zone with its head directed toward an open arm and allowed to explore the maze for 5 min. The time spent in the open arms was recorded with a camera and considered an index of an anxiolytic effect.

### Y maze

The Y maze testing procedure was performed as previously reported [27]. The Y maze apparatus consisted of 3 identical symmetrical arms (46 cm long × 3 cm wide × 17 cm high) with white acrylic walls. During an 8 min test session, each mouse was initially placed in the central space and allowed to freely explore in the Y maze. Arm entry was defined as the entry of all four paws into one arm. The spontaneous alternation percentage, an indicator of short-term memory, was calculated using the following equation: [the number of sequential triplets containing entries in the three arms/(total arm entries - 2) × 100].

### Morris water maze (MWM)

The MWM task was used to evaluate the spatial learning and memory abilities of mice as previously described [29]. The MWM assay consisted of four stages: pretraining (day 34), training (days 35–38), testing (day 39), and probe (day 40). The swimming ability of the mice was tested on day 34. Mice that floated in the pool were not included in the task. The MWM consisted of a large circular white pool (100 cm in diameter and 47 cm in height) filled with water (21 ± 2 °C) and divided into four quadrants. A hidden platform with a diameter of 10 cm was located 1 cm below the water level in the center of the target quadrant in the pool during the pretraining, training, and testing periods. The time to climb onto the hidden platform within 60 s was recorded as the escape latency. During days 35–38, mice were trained four times per day and allowed to rest for 15 min between trials. The mouse was allowed to search for the platform for 60 s and was allowed to remain on the platform for 20 s. If a mouse did not find the platform within 60 s, it was gently guided to the platform, where it remained for 20 s, and the escape latency was recorded as 60 s. The training curve from days 35 to 38 was recognized as an index of spatial learning ability. On day 39 (testing), three testing trials were conducted as an index of spatial learning acquisition. Twenty-four hours after the last testing trial, a probe test (day 40, two trials) was performed by removing the platform and allowing each mouse to swim freely for 60 s in the pool. The amount of time spent in the target quadrant was calculated as an index of long-term spatial memory.

### Analysis of fecal short chain fatty acid (SCFA) contents

Before sacrifice (on day 41), fresh feces (100 mg) were collected from individual mice (*n* = 5/group) and suspended in 1 ml of 0.5% phosphoric acid with 200 ppm 2-ethylbutyric acid as a surrogate standard. The fecal suspensions were homogenized by vortexing for 2 min and centrifuging for 10 min at 17000×g. The supernatant was extracted with an equal volume of ethyl acetate by vortexing for 10 min and centrifuging for 10 min at 17000×g. The organic extracts were stored at − 80 °C before analysis [30]. Prior to analysis, 600 μL of the organic extracts were transferred into a sample vial, and 4-methyl valeric acid was added as an internal standard at a final concentration of 200 ppm. A GC-MS analysis was performed using an Agilent 6890 N GC system equipped with an Agilent 5973 N mass detector (Agilent; Wilmington, Delaware, USA). A fused-silica capillary column DB-FFAP (30 m length, 0.25 mm inner diameter, 0.25 μm film thickness, J&W Scientific, Agilent) was used. Helium was supplied as the carrier gas at a rate of 1 ml/min. The injection was performed in splitless mode with an injection volume of 1 μL and an injector temperature of 250 °C. The initial oven temperature was maintained at 80 °C for 1 min, increased to 100 °C at 4 °C/min, maintained at 100 °C for 1 min, increased to 180 °C at 8 °C/min, maintained at 180 °C for 1 min, increased to 220 °C at 20 °C/min, and finally held at 220 °C for 5 min. The temperatures of the ion source, quadrupole, and interface were 230 °C, 150 °C, and 280 °C, respectively. The detector was operated in electron impact ionization mode (electron energy, 70 eV), scanning the 35–250 m/z range. The solvent delay was 3.5 min. SCFA mixtures consisting of acetic acid, propionic acid, and butyric acid were prepared at a series of concentrations (10, 20, 50, 100, 200, 300 and 400 ppm) with a 200 ppm internal standard to construct the calibration curves. The concentration of SCFAs was calculated from the internal standard curve.

### Immunohistochemistry (IHC)

Mice (*n* = 3/group) were transcardially perfused with saline followed by 4% paraformaldehyde in 0.1 M phosphate buffer (pH 7.4) under deep anesthesia induced by an intraperitoneal injection of avertin (0.4 g/kg; Sigma). The brains were transferred to a gradient of 10, 20, and 30% sucrose until they sank to the bottom of the container. Then, the brain samples were embedded in clear frozen sectioning compound (OCT, Sakura, USA) and cut into 30 μm coronal sections with a cryostat microtome (CMS3050S, Leica Microsystems; Nussloch, Germany). IHC was performed essentially as described in our recent report [29]. The free-floating sections (3–4 sections per mouse) were incubated with primary antibodies (Table 1) overnight at room temperature. After three washes with PBS, the sections were incubated with the appropriate biotinylated secondary antibodies (1:200, Vector Laboratories; CA, USA) for 1 h at room temperature, followed by an incubation with an avidin-biotin-peroxidase complex (ABC kit; Vector Laboratories) for 1 h at room temperature. The sections were washed with PBS (10 min, three times), and the signal was developed by a diaminobenzidine (DAB) kit (Vector Laboratories). After DAB staining, the slices were observed using a light microscope (Leica; Wetzlar, Germany). Images were obtained using Image-Pro Plus 5.1 software (Image-Pro Plus Media Cybernetics; Washington, MD, USA), and the threshold intensity was manually set according to a standard signal for all images. Pixel counts were derived from the average of three adjacent sections per animal.

**Table 1:** List of primary antibodies

Antibody	Species	Supplier	IHC dilution	WB dilution	Catalogue no.
Iba-1	Rabbit	Wako	1:1000		019–19,741
GFAP	Mouse	Millipore	1:1000		MAB360
NeuN	Mouse	Millipore	1:3000		MAB377
ChAT	Rabbit	COVANCE	1:1000		AB143
5-HT	Rat	Millipore	1:200		MAB352
TH	Rabbit	Millipore	1:1000		AB152
6E10	Mouse	Biolegend		1:1000	SIG-39320
AβPP	Rabbit	Sigma-Aldrich		1:1000	SAB3500274
Beta-secretase 1 (BACE1)	Rabbit	Cell Signaling		1:1000	5606
Insulin-degrading enzyme (IDE)	Mouse	Abcam		1:1000	Ab32216
Neprilysin (NEP)	Mouse	Abcam		1:1000	Ab81688
PSD95	Rabbit	Abcam		1:1000	Ab18258
synaptophysin	Rabbit	Abcam		1:1000	Ab14692
GSK3β	Rabbit	Cell Signaling		1:1000	9315S
GSK3β (pS9)	Rabbit	Cell Signaling		1:1000	9336
Tau (pS202)	Rabbit	Anaspec		1:1000	AS-28017
Tau (pT231)	Rabbit	Invitrogen		1:1000	44746G
HT7	Mouse	Thermo		1:1000	MN1000
GAPDH	Mouse	Arigobio		1:1000	ARG10112

*WB* Western blot, *IHC* Immunohistochemistry

### Western blot analyses

Western blot analyses were completed as previously described [29]. Briefly, hippocampal samples were collected (*n* = 5–8 mice/group) and homogenized in RIPA lysis buffer (1:2, w/v). A bicinchoninic acid (BCA) protein assay kit (Pierce, Rockford, USA) was utilized to determine the protein concentration. Subsequently, 25 μg of protein were loaded and separated on SDS-PAGE gels and transferred to a polyvinylidene fluoride (PVDF) membrane (Millipore). The membranes were blocked with TBS containing 0.05% Tween 20 (TBST) and 5% skim milk for 1 h at 37 °C and then incubated with primary antibodies (Table 1) for 16 h at 4 °C. After washes with TBST, the membranes were incubated with the secondary antibodies (1:10000, Amersham Pharmacia Biotech; Piscataway, NJ, USA) corresponding to the type of primary antibody for 1 h at room temperature. Glyceraldehyde-3-phosphate dehydrogenase (GAPDH) was used as a loading control. The membranes were developed using an enhanced chemiluminescence (ECL) detection reagent kit (Amersham Pharmacia Biotech) and visualized using an LAS-4000 chemiluminescence detection system (Fujifilm; Tokyo, Japan). Band densities were quantified using Gel-Pro Analyzer software (GelPro32, version 4.0; Media Cybernetics, Inc.; Rockville, MD, USA).

### Statistical analysis

The data are presented as the means ± standard errors of the means (SEM), and the statistical analysis was performed using one-way analysis of variance (ANOVA) followed by a post hoc least significant difference (LSD) test. Statistical and data analyses were carried out with IBM SPSS software 20.0 (SPSS; Chicago, IL, USA). A *P* value of 0.05 or less was considered significant.

## Results

### PS128 supplementation prevented cognitive dysfunction in 3 × Tg-AD mice administered icv-STZ

**Fig. 1:**
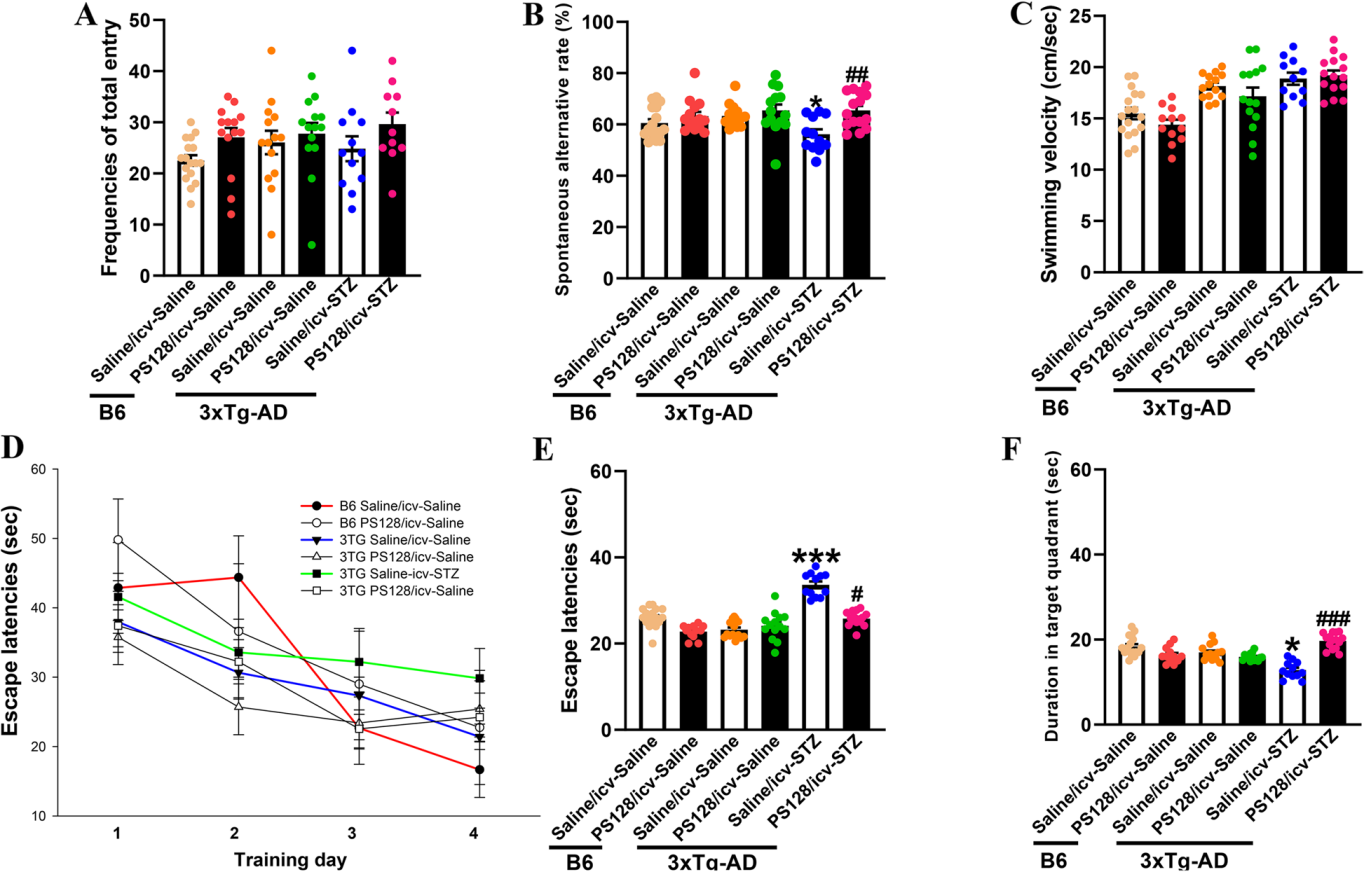
The effects of PS128 supplementation on the cognitive function of 3 × Tg-AD mice treated with icv-STZ. **A** The total entry frequencies of mice in the Y maze were not different among the groups, which indicated no specific preferences for any the arms among the mice. **B** The spontaneous alternation rate of 3 × Tg-AD and B6 mice treated with PS128 or/and icv-STZ. For 3 × Tg-AD mice, short-term memory retrieval was better in the PS128/icv-STZ group than in the saline/icv-STZ group. **C** Swimming velocity of the mice in the MWM. No difference in the swimming ability of the mouse groups was observed. **D** Learning curves of the mice over the 4 training days of the MWM. PS128 prevented the retarded learning curve induced by icv-STZ in 3 × Tg-AD mice during the training period. **E** Spatial learning acquisition in the testing phase. PS128 prevented the impairment of spatial learning acquisition induced by icv-STZ in 3 × Tg-AD mice. **F** The long-term memory retrieval results. A probe trial was conducted 24 h after the last testing trial to evaluate long-term memory retrieval. PS128 prevented the impairment in long-term memory retrieval induced by icv-STZ in 3 × Tg-AD mice. The data are presented as the means ± SEM (*n* = animals 15–20/group). * *P* < 0.05 and *** *P* < 0.001 compared with the 3 × Tg-AD mice in the saline/icv-saline group. # *P* < 0.05, ## *P* < 0.01, and ### *P* < 0.001 compared with the 3 × Tg-AD mice in the saline/icv-STZ group

No differences identified in the blood glucose and anxiety levels were observed among all groups (Fig. S1B-E). We measured short-term memory with the Y maze and spatial cognition with the MWM to further investigate the ability of the PS128 pretreatment to protect against the cognitive dysfunction induced by icv-STZ in mice. Based on the results of the Y maze, the administration of PS128 or/and icv-STZ did not affect the total entry frequency, indicating that all groups of mice had the same ability to explore novel environments (Fig. 1A). Subsequently, the spontaneous alternation rate was decreased in the icv-STZ group compared to the icv-saline group of 3 × Tg-AD mice (*P* < 0.05, Fig. 1B). However, pretreatment with PS128 significantly prevented the decrease in the spontaneous alternation rate in 3 × Tg-AD mice treated with icv-STZ (*P* < 0.01, Fig. 1B). The results indicate that the PS128 pretreatment prevented the short-term memory deficit induced by icv-STZ in 3 × Tg-AD mice. In addition to short-term memory, we further analyzed spatial learning and memory with the MWM test. First, we did not observe a significant difference in swimming ability among all groups of mice, suggesting that all groups of mice had the same level of motor activity (Fig. 1C). From the results of the learning curve, the time required to climb onto the hidden platform (escape latency) was decreased following training in B6 (F(3, 30) = 6.68, *P* < 0.001; Fig. 1D) and 3 × Tg-AD (F(3, 55) = 4.45, *P* < 0.01; Fig. 1D) mice in the saline/icv-saline group. However, the escape latency was not significantly reduced following training in 3 × Tg-AD mice treated with saline/icv-STZ (F(3, 39) = 1.641, *P* > 0.05; Fig. 1D). The PS128 pretreatment prevented the impaired learning curve in 3 × Tg-AD mice induced by icv-STZ (F(3, 59) = 4.975, *P* < 0.01; Fig. 1D). For spatial learning acquisition (testing), the escape latencies were not significantly different between B6 and 3 × Tg-AD mice in the saline/icv-saline group (*P* > 0.05; Fig. 1E). However, 3 × Tg-AD mice in the saline/icv-STZ group required more time to reach the platform (escape latency) than mice in the saline/icv-saline group (*P* < 0.001; Fig. 1E), and PS128 significantly prevented the impairment in spatial learning acquisition induced by icv-STZ (*P* < 0.05; Fig. 1E). In the probe trials, the time spent in the target quadrant was not significantly different between B6 and 3 × Tg-AD mice in the saline/icv-saline group (*P* > 0.05; Fig. 1F). However, the length of time spent in the target quadrant was significantly decreased in 3 × Tg-AD mice in the saline/icv-STZ group compared with those in the saline/icv-saline group (*P* < 0.05; Fig. 1F). On the other hand, PS128 prevented the decrease in the length of time spent in the target quadrant by 3 × Tg-AD mice treated with icv-STZ (*P* < 0.001; Fig. 1F). Taken together, the PS128 pretreatment prevented the cognitive dysfunction induced by icv-STZ in 3 × Tg-AD mice.

### PS128 supplementation prevented the increase in GSK3β activity and Aβ production in 3 × Tg-AD mice treated with icv-STZ

**Fig. 2:**
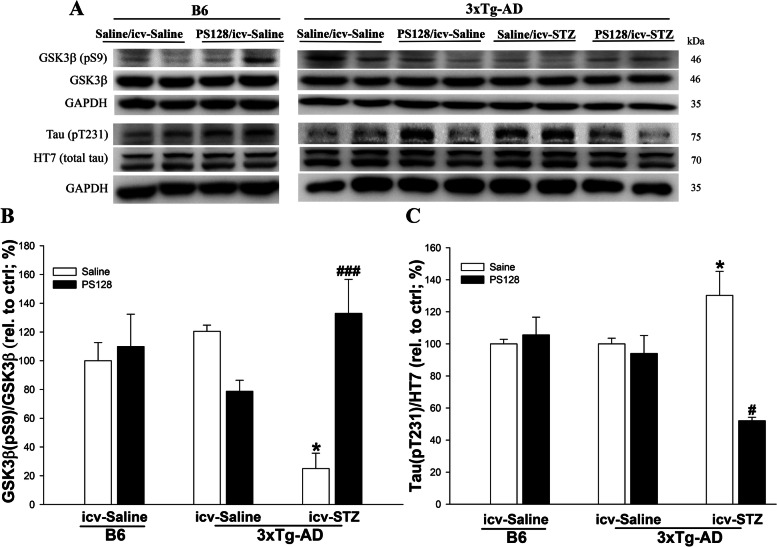
The effects of PS128 supplementation on the levels of the phosphorylated tau protein and other related proteins in 3 × Tg-AD mice treated with icv-STZ. **A** Representative image of western blots. **B** and **C** Quantitative densitometry results of the ratios of GSK3β (pS9)/GSK3β and pT231/HT7 (total tau). GAPDH served as the internal control. The quantitative data are presented as the means ± SEM (*n* = 5–8 animals/group). * *P* < 0.05 compared with the 3 × Tg-AD mice in the saline/icv-saline group. # *P* < 0.05 and ### *P* < 0.001 compared with the 3 × Tg-AD mice in the saline/icv-STZ group

**Fig. 3:**
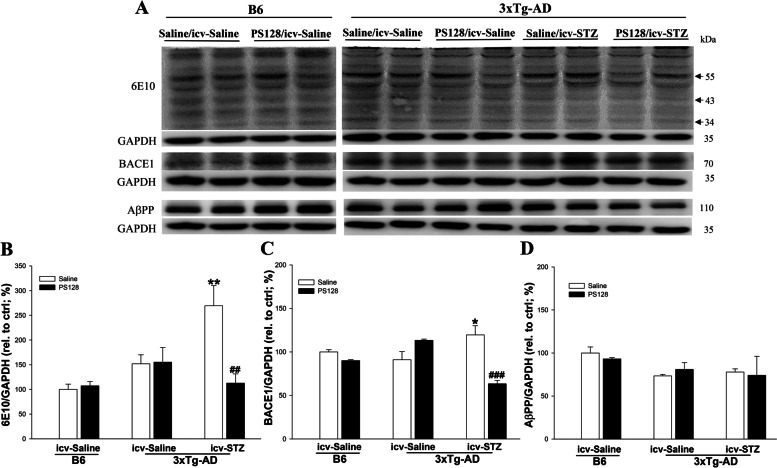
The effects of PS128 supplementation on the levels of Aβ deposition-related proteins in 3 × Tg-AD mice treated with icv-STZ. **A** Representative image of western blots. **B**-**D** Quantitative densitometry results for the ratios of 6E10/GAPDH, BACE1/GAPDH, and AβPP/GAPDH. GAPDH served as the internal control. The quantitative data are presented as the means ± SEM (*n* = 5–8 animals/group). * *P* < 0.05 and ** *P* < 0.05 compared with the 3 × Tg-AD mice in the saline/icv-saline group. ## *P* < 0.01 and ### *P* < 0.001 compared with the 3 × Tg-AD mice in the saline/icv-STZ group

The early administration of icv-STZ induces cognitive dysfunction by increasing GSK3β activity and levels of the phosphorylated tau protein [31]. Therefore, the levels of inactive GSk3β (pS9) and tau protein phosphorylation at T231 (pT231) and S202 (pS202) sites were measured in this study. In 3 × Tg-AD mice, the levels of GSK3β (pS9) were significantly decreased in the saline/icv-STZ group compared to the saline/icv-saline group (*P* < 0.05; Fig. 2A-B). However, the PS128 pretreatment significantly prevented the decrease in GSK3β (pS9) activation induced by icv-STZ in 3 × Tg-AD mice (*P* < 0.01; Fig. 2A-B). The levels of pT231, but not pS202, were significantly increased in 3 × Tg-AD mice treated with saline/icv-STZ compared to saline/icv-saline mice (*P* < 0.05; Fig. 2A and C and Fig. S2A-B), and PS128 supplementation prevented the increase in levels the pT231 tau protein (*P* < 0.05; Fig. 2A and C). In addition, the levels of GSK3β (pS9) and phosphorylated tau proteins were not significantly different between B6 and 3 × Tg-AD mice treated with saline/icv-saline conditions (*P* > 0.05; Fig. 2A-C and Fig. S2A-B). Except the changes in GSK3β activity and tau pT231 phosphorylation levels, the abnormal deposition of Aβ produced by the sequential cleavage of β-site AβPP-cleaving enzyme (BACE1) and γ-secretase from AβPP is a major pathological feature of AD [32]. Evidence has also suggested that an imbalance in Aβ metabolic enzymes, including BACE1 for Aβ production, insulin-degrading enzyme (IDE) and neprilysin (NEP) for Aβ clearance, causes Aβ deposition [33, 34]. Therefore, the levels of the 6E10, BACE1, AβPP, IDE, and NEP proteins in the mouse hippocampus were examined using western blot analysis. Based on the immunoblot results, we did not observe significant differences in levels of the 6E10, BACE1, APP, IDE, and NEP proteins between B6 and 3 × Tg-AD mice in the saline/icv-saline group (*P* > 0.05; Fig. 3 and Fig. S2A, C and D). The levels of 6E10 (*P* < 0.01; Fig. 3A-B) and BACE1 (*P* < 0.05; Fig. 3A and C) were significantly increased in 3 × Tg-AD mice treated with saline/icv-STZ compared to mice treated with saline/icv-saline. PS128 prevented the significant increase in 6E10 (*P* < 0.01; Fig. 3A-B) and BACE1 (*P* < 0.001; Fig. 3A and C) protein levels induced by icv-STZ in 3 × Tg-AD mice. However, significant changes in the levels of APP, IDE, and NEP proteins were not observed in all mouse groups (*P* > 0.05; Fig. 3A and D and Fig. S2A, C, and D). These data suggest that PS128 prevented Aβ deposition mediated by BACE1 and GSK3β (pS9) to protect against the toxicity induced by icv-STZ in 3 × Tg-AD mice.

### PS128 supplementation reduced the fecal propionic acid (PPA) levels in 3 × Tg-AD mice treated with icv-STZ

**Fig. 4:**
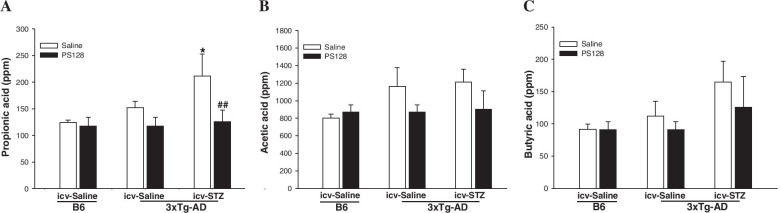
The effects of PS128 supplementation on the fecal SCFA levels in 3 × Tg-AD mice treated with icv-STZ. **A** The levels of PPA in fecal boli. The administration of icv-STZ increased the fecal PPA levels, while PS128 prevented this increase. **B** and **C** The levels of acetic acid and butyric acid in fecal boli. The administration of PS128 or/and icv-STZ had no effects on the levels of fecal acetic acid and butyric acid in mice. The quantitative data are presented as the means ± SEM (*n* = 5 per group). * *P* < 0.05 compared with the 3 × Tg-AD mice in the saline/icv-saline group. ## *P* < 0.01 compared with the 3 × Tg-AD mice in the saline/icv-STZ group

The gas chromatography-mass spectrometry analysis of SCFAs revealed that the levels of PPA, acetic acid, and butyric acid were not different between 3 × Tg-AD mice and B6 mice treated with saline/icv-saline (Fig. 4). The administration of icv-STZ significantly increased the fecal PPA levels in 3 × Tg-AD mice (*P* < 0.05; Fig. 4A). In addition, PS128 supplementation prevented the increase in fecal PPA levels induced by icv-STZ in 3 × Tg-AD mice (*P* < 0.01; Fig. 4A). However, the administration of PS128 and/or icv-STZ did not alter the fecal acetic acid and butyric acid levels in 3 × Tg-AD mice (Fig. 4B-C). Therefore, we suggest that PS128 supplementation prevented cognitive dysfunction in icv-STZ-treated 3 × Tg-AD mice potentially through the modulation of PPA levels, but not acetic acid or butyric acid levels.

### PS128 supplementation prevented gliosis in 3 × Tg-AD mice treated with icv-STZ

**Fig. 5:**
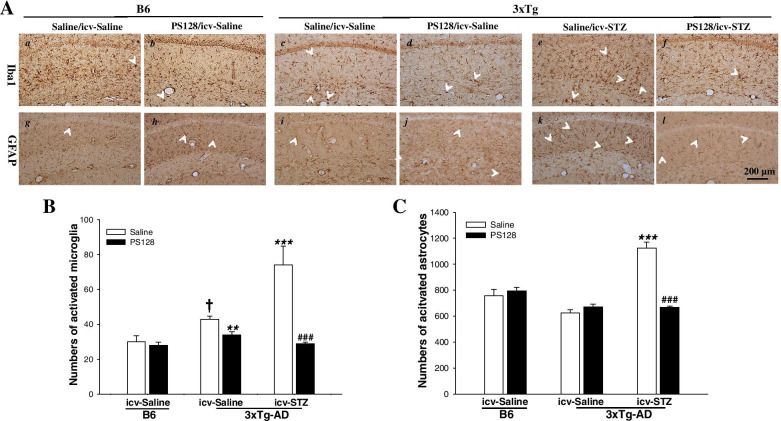
The effects of PS128 supplementation on the levels of gliosis in 3 × Tg-AD mice treated with icv-STZ. **A** Representative images of immunostaining of microglia with Iba1 antibodies and astrocytes with GFAP antibodies in the hippocampus of different groups. The quantitative results for activated microglia (**B**) and astrocytes (**C**). Scale bar = 200 μm. Arrowheads, positive staining signals. The quantitative data are presented as the means ± SEM (*n* = 3 animals per group). † *P* < 0.05 compared with the B6 mice in the saline/icv-saline group. ** *P* < 0.05 and *** *P* < 0.001 compared with the 3 × Tg-AD mice in the saline/icv-saline group. ### *P* < 0.001 compared with the 3 × Tg-AD mice in the saline/icv-STZ group

The activation of astrocytes and microglia aggravates the neurodegenerative milieu through the release of proinflammatory mediators, which results in cognitive impairment [35, 36]. We performed staining with antibodies against glial fibrillary acidic protein (GFAP) and Iba1 to assess astrogliosis and microgliosis, respectively. The immunostaining results revealed significantly increased numbers of activated microglia, but not astrocytes, in 3 × Tg-AD mice compared to B6 mice treated with saline/icv-saline (*P* < 0.05; Fig. 5A-B). In 3 × Tg-AD mice, the gliosis of astrocytes and microglia was significantly increased in the saline/icv-STZ group compared to the saline/icv-saline group (*P* < 0.001; Fig. 5A-C). However, PS128 prevented the gliosis induced by icv-STZ in 3 × Tg-AD mice (*P* < 0.001; Fig. 5A-C). In addition, in 3 × Tg-AD mice, microgliosis was significantly decreased in the PS128/icv-saline group compared to the saline/icv-saline group (*P* < 0.01; Fig. 5A-B). Based on these data, PS128 prevented icv-STZ-induced gliosis in 3 × Tg-AD mice.

### PS128 supplementation prevented the decrease in PSD95 and synaptophysin expression levels in the 3 × Tg-AD mice treated with icv-STZ

**Fig. 6:**
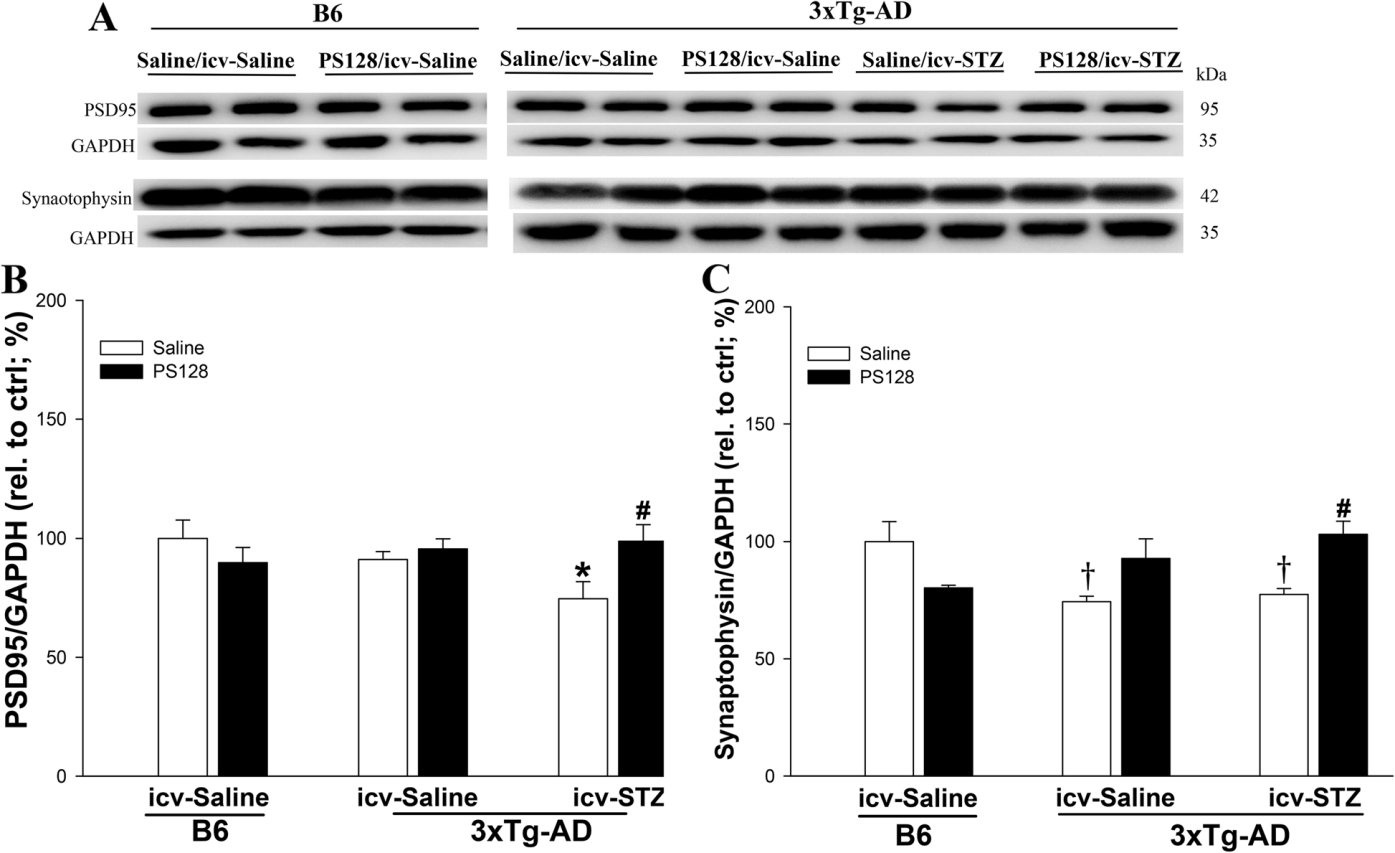
The effects of PS128 supplementation on the levels of PSD95 and synaptophysin in 3 × Tg-AD mice treated with icv-STZ. **A** Representative image of western blots. **B** and **C** Quantitative densitometry results of PSD95 and synaptophysin expression relative to the control, with GAPDH serving as the internal control. The quantitative data are presented as the means ± SEM (*n* = 5–8 animals per group). † *P* < 0.05 compared with the B6 mice in the saline/icv-saline group.* *P* < 0.05 compared with the 3 × Tg-AD mice in the saline/icv-saline group. # *P* < 0.05 compared with the 3 × Tg-AD mice in the saline/icv-STZ group

Synaptic proteins, including postsynaptic density protein 95 (PSD95) and synaptophysin, play critical roles in synaptic plasticity and cognitive function [37, 38]. Therefore, the levels of PSD95 and synaptophysin proteins were examined using western blot analyses. The level of synaptophysin, but not that of PSD95, was significantly reduced in 3 × Tg-AD mice compared to B6 mice treated with saline/icv-saline (*P* < 0.05; Fig. 6). In 3 × Tg-AD mice, the PSD95 expression level was significantly decreased after treatment with saline/icv-STZ compared to animals treated with saline/icv-saline (*P* < 0.05; Fig. 6A-B). However, PS128 supplementation prevented the decrease in PSD95 (*P* < 0.05; Fig. 6A-B) and synaptophysin (*P* < 0.01; Fig. 6A and C) expression levels caused by icv-STZ in 3 × Tg-AD mice. Thus, PS128 supplementation in 3 × Tg-AD mice prevented the decreases in the levels of the synaptic proteins PSD95 and synaptophysin induced by icv-STZ.

### PS128 supplementation prevented the loss of neurons related to cognitive function in 3 × Tg-AD mice treated with icv-STZ

**Fig. 7:**
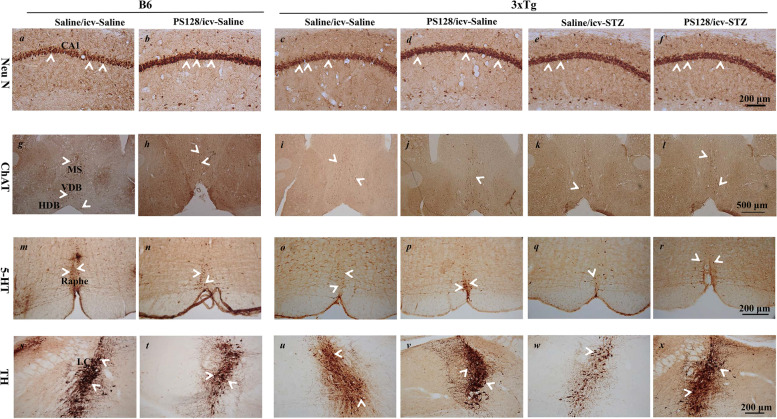
The effects of PS128 supplementation on cognition-related neurons in 3 × Tg-AD mice treated with icv-STZ. Representative images of immunostaining of mature pyramidal neurons with NeuN antibodies in the CA1 subregion of the hippocampus (*a*-*f*), cholinergic neurons with ChAT antibodies in the MS/DB (*g*-*l*), serotonergic neurons in the raphe nucleus (*m*-*r*), and noradrenergic neurons in the LC region (*s*-*x*) of different groups. Scale bars = 200 μm for NeuN, 5-HT, and TH and 500 μm for ChAT. Arrowheads, positive staining signals. Mature neuron (NeuN), serotonergic neuron (5-HT), noradrenergic neuron (TH), cholinergic neuron (ChAT), medial septum/diagnoid band (MS/DB), and locus coeruleus (LC)

The number of neurons related to cognition was evaluated in this study to determine whether PS128 or/and icv-STZ affected pyramidal neurons in the CA1 region of the hippocampus, cholinergic neurons in the medial septum/diagnoid band (MS/DB), serotonergic neurons in the raphe nucleus, and noradrenergic neurons in the locus coeruleus (LC). Based on the immunostaining results (Fig. 7 and Table 2), only the number of cholinergic neurons was significantly decreased in 3 × Tg-AD mice compared to B6 mice treated with saline/icv-saline (*P* < 0.001). In addition, in 3 × Tg-AD mice, the loss of pyramidal neurons (*P* < 0.01), cholinergic neurons (*P* < 0.001), serotonergic neurons (*P* < 0.001), and noradrenergic neurons (*P* < 0.01) was significantly increased in the saline/icv-STZ group compared to the saline/icv-saline group. However, PS128 supplementation prevented the loss of pyramidal neurons (*P* < 0.01), cholinergic neurons (*P* < 0.05), serotonergic neurons (*P* < 0.01), and noradrenergic neurons (*P* < 0.001), protecting against the damage induced by icv-STZ in 3 × Tg-AD mice. These results show that PS128 supplementation prevented the loss of neurons related to cognitive function in 3 × Tg-AD mice treated with icv-STZ.

**Table 2:** Quantification of the numbers of cognition-related neurons in mice

	B6		3xTg-AD			
	icv-saline		icv-saline		icv-STZ	
	Saline	PS128	Saline	PS128	Saline	PS128
**NeuN**	1299.8 ± 119.4	1576.6 ± 98.1	1356.5 ± 157.2	1266.3 ± 81.3	784.3 ± 109.7^**^	1305.2 ± 64.0^*##*^
**ChAT**	830.3 ± 68.7	944.7 ± 24.1	547.5 ± 13.3^*†††*^	491.0 ± 15.3	234.0 ± 36.5^***^	390.7 ± 60.4^*#*^
**5-HT**	136.0 ± 17.2	107.5 ± 7.8	63.3 ± 29.2	125.3 ± 7.1	46.0 ± 1.6^***^	118.3 ± 27.8^*##*^
**TH**	324.9 ± 22.3	271.0 ± 31.8	289.8 ± 33.2	266.5 ± 22.5	162.8 ± 9.2^**^	296.7 ± 25.5^*###*^

^†^Compared to B6 mice in the icv-saline/saline group

^*^Compared to 3xTg-AD mice in the icv-saline/saline group

^#^Compared to 3xTg-AD mice in the icv-STZ/saline group

^†††^*P* < 0.001

**P < 0.01; ***P < 0.001

^#^*P* < 0.05; ^##^*P* < 0.01; ^###^*P* < 0.001

## Discussion

According to our findings, the administration of icv-STZ exacerbated the disease progression of AD via gliosis that resulted from an increases in the levels of fecal PPA and the inactive form of GSK3β in 3 × Tg-AD mice. However, PS128 supplementation prevented the deleterious effects of icv-STZ on 3 × Tg-AD mice. In addition, we did not observe a difference in cognitive function between B6 wild-type and 3 × Tg-AD mice, as only a few changes in microgliosis, synaptophysin protein expression levels, and cholinergic neurons were observed at 7 months of age. Furthermore, PS128 supplementation alone did not exert significant toxic effects on B6 wild-type mice and only decreased the level of the number of activated microglia in the hippocampus of naïve 3 × Tg-AD mice. Gliosis resulting from PPA and GSK3β was considered one of the possible mechanisms that attenuated disease progression in the AD mouse model. Therefore, PS128 supplementation is a safe and potential preventive strategy for AD progression.

In 3 × Tg-AD mice, the administration of icv-STZ induced cognitive dysfunction resulting from gliosis by increasing the levels of GSK3β activity and PPA. In addition, gliosis was associated with increased Aβ deposition, phosphorylation of tau at site T231 (pT231), and BACE1 levels and decreased levels of the postsynaptic protein PSD95 and numbers of cognition-related neurons, including pyramidal neurons in the CA1 subregion of the hippocampus, cholinergic neurons in the MS/DB, serotonergic neurons in the raphe nucleus, and noradrenergic neurons in the LC. These findings of the progression of cognitive dysfunction induced by icv-STZ in 3 × Tg-AD mice are consistent with many other studies [27, 39, 40]. In addition, the administration of icv-STZ triggers insulin resistance and then impairs insulin signaling in the brain [41], but the peripheral glucose level was not changed in 3 × Tg-AD mice, as shown in previous study [23]. Insulin resistance is associated with the modulation of amyloid production, tau phosphorylation and neuroinflammation through the regulation of GSK3β activity [42, 43]. Furthermore, GSK3β activation subsequently leads to a number of changes, among which tau hyperphosphorylation [44] and increased Aβ production by activated BACE1 [45] play a significant role in AD pathogenesis. The phosphorylation of tau at site T231 precedes phosphorylation at the S202 site in individuals with AD [46, 47], and pT231 was the primary site phosphorylated by GSK3β [48]. In addition, the administration of icv-STZ to 3 × Tg-AD mice increased the expression levels of BACE1, but not AβPP, IDE or NEP. Many studies have also suggested that icv-STZ increases GSK3β activity and subsequently regulates Aβ deposition by modulating BACE1 expression [27, 31, 49, 50]. The substantially increased deposition of Aβ might be mediated by the regulation of both BACE1 and GSK3β activity in the 3 × Tg-AD mice treated with icv-STZ. The deposition of Aβ and phosphorylated tau protein triggers a series of reactions, including gliosis and synaptic dysfunction, resulting in neurodegeneration [51–53]. Accumulating evidence has also indicated that excess PPA crosses the blood-brain barrier and then induces gliosis [54, 55]. A reduction in the neuron number and increase in gliosis were identified in the PPA-treated brain [56]. Excess PPA exerts many negative effects, including gliosis and ASDs [55, 57–59]. The association described above indicates that increased GSK3β activity and fecal PPA levels might increase gliosis. A previous meta-analysis also reported the loss of cholinergic neurons in the nucleus basalis, serotonergic neurons in the raphe nucleus, and noradrenergic neurons in the LC of patients with AD [60, 61]. Based on the combination of the genetic (3 × Tg-AD mice) and environmental (the type 3 AD) factors, and the phenotypic features described above, icv-STZ administration to 3 × Tg-AD mice was proven to be a reliable animal model to evaluate the effects and mechanisms of PS128 in AD mice.

Results from a recent randomized, double-blind, placebo-controlled trial of younger (aged 7–15) individuals with autism spectrum disorder (ASD) in Taiwan showed that PS128 ameliorates opposition/defiance behaviors [62]. PS128 also exerts anxiolytic effects on stressed mice [16, 17]. In addition, PS128 is safe for human consumption, according to toxicological assessments [63]. In this study, we assessed the effect of PS128 on the icv-STZ-treated 3 × Tg-AD model and showed that long-term supplementation with PS128 prevented cognitive dysfunction induced by icv-STZ in the 3 × Tg-AD male mice. The beneficial effects of PS128 supplementation on attenuating cognitive deficits were consistent with many other probiotic studies [14, 64–67]. Therefore, we suggest that PS128 supplementation is safe and has the potential to prevent cognitive dysfunction in individuals with AD.

**Fig. 8:**
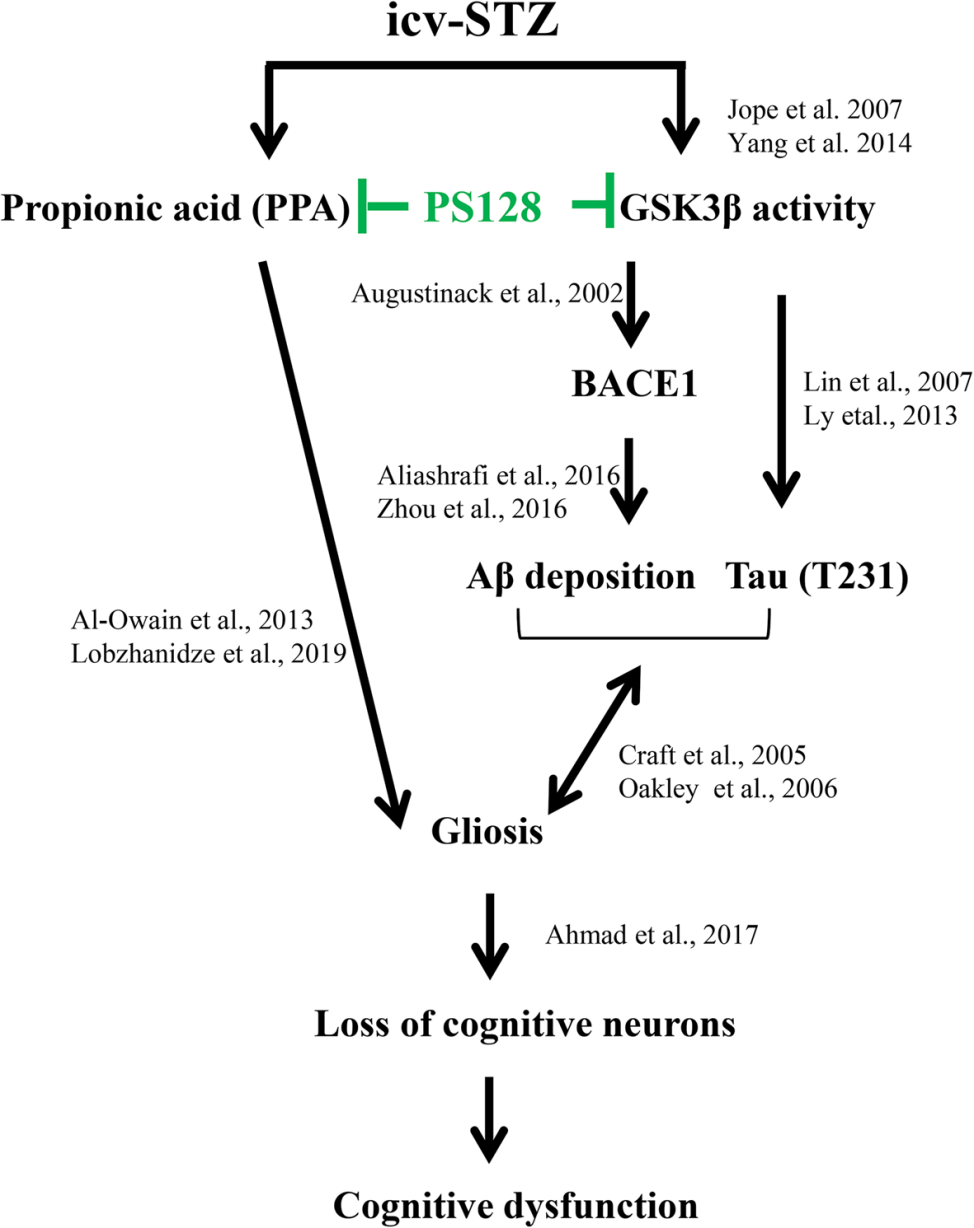
The proposed effect of PS128 supplementation on 3 × Tg-AD mice treated with icv-STZ. The icv-STZ treatment substantially increased gliosis by increasing GSK3β activity and fecal PPA levels, resulting in cognitive dysfunction in 3 × Tg-AD mice. However, PS128 supplementation prevented the damage induced by icv-STZ, possibly by reducing PPA levels, GSK3β activity, and gliosis in 3 × Tg-AD mice, which further ameliorated the behavioral and pathological features of AD

PS128 supplementation alters hyperactive and emotional behaviors by modulating the levels of monoamine neurotransmitters and systemic inflammation [16, 17, 68]. Researchers have not clearly determined whether changes in metabolites such as SCFAs, the main metabolites of gut microbiota, are modulated by the beneficial effect of PS128 on AD. SCFAs are often considered critical candidate mediators of gut-brain communication, and altered SCFA production has been reported in a variety of neuropathologies [69]. In the current study, PS128 supplementation prevented the increase in fecal PPA levels induced by icv-STZ in 3 × Tg-AD mice. The results seem inconsistent with some previous evidence suggesting that SCFAs, including acetic acid, butyric acid, and PPA, exerted positive effects on individuals with AD [70, 71]. However, the results of a recent bioinformatics analysis indicated that PPA specifically plays an important role in AD pathogenesis [54]. PPA levels also play an important role in insulin resistance [72] and are increased in subjects with type 2 diabetes mellitus [73] due to alterations in the environment of the gut microbiota [74, 75]. PPA readily crosses the gut–blood barrier and then enters the central nervous system to impair spatial cognitive behavior [76–78]. Furthermore, probiotics restore the normal gut microbiota and protect against damage induced by PPA [79]. Probiotics significantly improve the cognitive function of 3 × Tg-AD mice by increasing neuronal activity, attenuating gliosis, mitigating synaptic deficits, and reducing the levels of phosphorylated tau though the inhibition of GSK3β activity [80]. Therefore, the modulation of the levels of PPA and GSK3β activity by PS128 supplementation may play a role in preventing cognitive dysfunction and promoting memory consolidation in the 3 × Tg-AD mice treated with icv-STZ, as shown in Fig. 8. However, further studies are needed to elucidate whether the other mechanisms mediating the beneficial effects of PS128 on cognition are involved.

## Conclusions

In summary, the administration of icv-STZ substantially increased levels of gliosis resulting from increased GSK3β activity and fecal PPA levels in 3 × Tg-AD mice. PS128 supplementation prevented the damage induced by icv-STZ by reducing the levels of fecal PPA, GSK3β activity, and gliosis in 3 × Tg-AD mice. Therefore, the PS128 supplementation represents a potential strategy to prevent and/or delay the progression of AD.

## Supplementary Information

**Additional file 1: Figure S1.** The effects of PS128 supplementation on anxiety behavior in 3 × Tg-AD mice treated with icv-STZ. **Figure S2.** The effects of PS128 supplementation on the levels of tau (pS202), IDE, and NEP protein expression in 3 × Tg-AD mice treated with icv-STZ. **Figure S3.** Original Uncropped Western blots for GSK3β related protein. **Figure S4.** Original Uncropped Western blots for phosphorylated Tau protein. **Figure S5.** Original Uncropped Western blots for 6E10 protein. **Figure S6.** Original Uncropped Western blots for BACE1 protein. **Figure S7.** Original Uncropped Western blots for AβPP protein. **Figure S8.** Original Uncropped Western blots for PSD95 protein. **Figure S9.** Original Uncropped Western blots for synaptophysin protein.

## Data Availability

The dataset used and/or analyzed during the current study are available from the corresponding author upon reasonable request.

## Abbreviations

PS128: *Lactobacillus plantarum* PS128
AD: Alzheimer’s disease
CFU: Colony-forming unit
icv: Intracerebroventricular
STZ: Streptozotocin
o.g.: Oral gavage
Aβ: Amyloid-β
NFTs: Neurofibrillary tangles
AβPP: Amyloid-β protein precursor
BACE1: β-site AβPP-cleaving enzyme
GSK3β: Glycogen synthase kinase 3 beta
SCFAs: Short-chain fatty acids
PPA: Propionic acid levels
PSD95: Postsynaptic density protein 95
MS/DB: Medial septum/diagnoid band
ChAT: Choline acetyltransferase
OFT: Open field test
EPM: Elevated plus maze
MWM: Morris water maze
IHC: Immunohistochemistry
DAB: Diaminobenzidine
GFAP: Glial fibrillary acidic protein
Iba1: Ionized calcium binding adaptor molecule 1
LC: Locus coeruleus
TH: Thyroxine hydroxylase
5-HT: 5-hydroxytryptamine
PVDF: Polyvinylidene fluoride
GAPDH: Glyceraldehyde-3-phosphate dehydrogenase
IDE: Insulin-degrading enzyme
NEP: Neprilysin
