# Correction: *Lactobacillus plantarum* PS128 prevents cognitive dysfunction in alzheimer’s disease mice by modulating propionic acid levels, glycogen synthase kinase 3 beta activity, and gliosis

**DOI:** 10.1186/s12906-022-03596-z

**Published:** 2022-05-17

**Authors:** Hei-Jen Huang, Jie-Ling Chen, Jian-Fu Liao, Yu-Hsin Chen, Min-Wei Chieu, Ya-Yun Ke, Chih-Chieh Hsu, Ying-Chieh Tsai, Hsiu Mei Hsieh-Li

**Affiliations:** 1 Department of Nursing, MacKay Junior College of Medicine, Nursing and Management, Taipei, 11260 Taiwan; 2 Department of Life Science, National Taiwan Normal University, Taipei, 11677 Taiwan; 3 Institute of Biochemistry and Molecular Biology, National Yang Ming Chiao Tung University, Taipei, 11221 Taiwan; 4 Bened Biomedical Co., Ltd., Taipei, 10448 Taiwan

**Correction: BMC Complement Med Ther 21, 259 (2021)**

**https://doi.org/10.1186/s12906-021-03426-8**

Following publication of the original article [1], the authors identified errors in Figs. 3(A), 7(e & k) in the article and Fig. S6 in the Additional file 1. The correct Figs. 3(A), 7(e & k), and Fig. S6(c-d) are shown in the revised version as following:

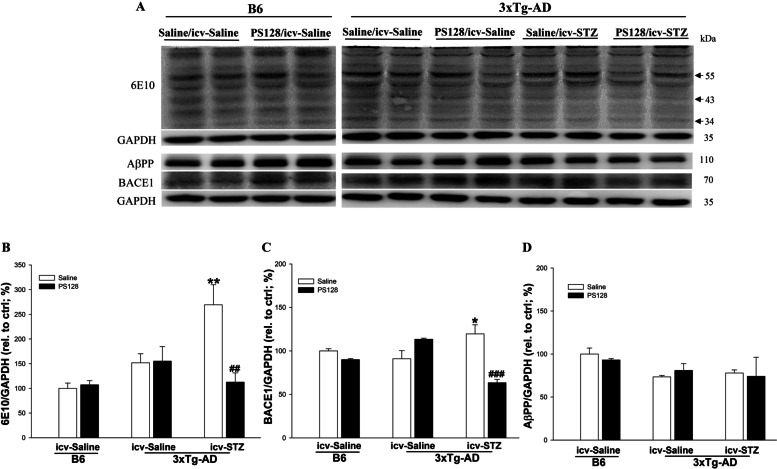



Fig. 3

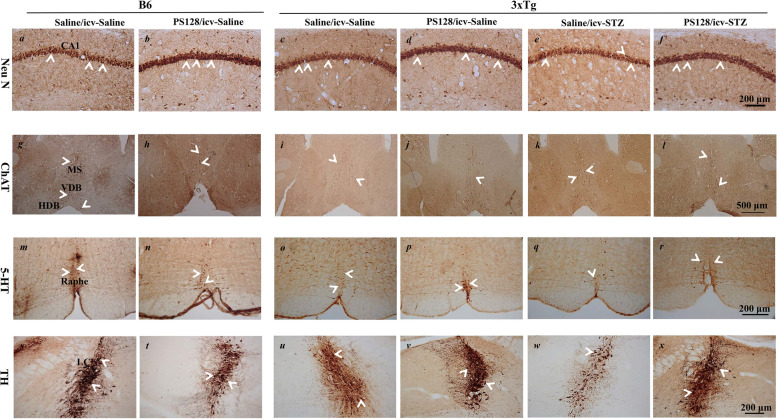



Fig. 7

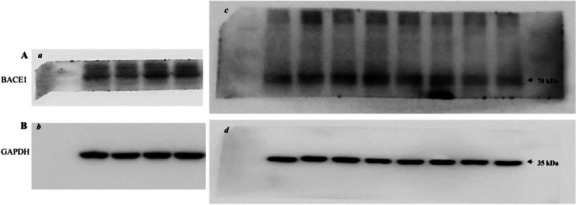



Fig. S6
