# Supplementary material for: Lactobacillus plantarum PS128 prevents cognitive dysfunction in Alzheimer’s disease mice by modulating propionic acid levels, glycogen synthase kinase 3 beta activity, and gliosis
Source: BMC Complement Med Ther. 2021 Oct 9;21:259. doi: 10.1186/s12906-021-03426-8 (PMC8502419; doi:10.1186/s12906-021-03426-8)
Supplement: Supplementary file 1 — Additional file 1: Figure S1. The effects of PS128 supplementation on anxiety behavior in 3 × Tg-AD mice treated with icv-STZ. Figure S2. The effects of PS128 supplementation on the levels of tau (pS202), IDE, and NEP protein expression in 3 × Tg-AD mice treated with icv-STZ. Figure S3. Original Uncropped Western blots for GSK3β related protein. Figure S4. Original Uncropped Western blots for phosphorylated Tau protein. Figure S5. Original Uncropped Western blots for 6E10 protein. Figure S6. Original Uncropped Western blots for BACE1 protein. Figure S7. Original Uncropped Western blots for AβPP protein. Figure S8. Original Uncropped Western blots for PSD95 protein. Figure S9. Original Uncropped Western blots for synaptophysin protein. [file 12906_2021_3426_MOESM1_ESM.docx]

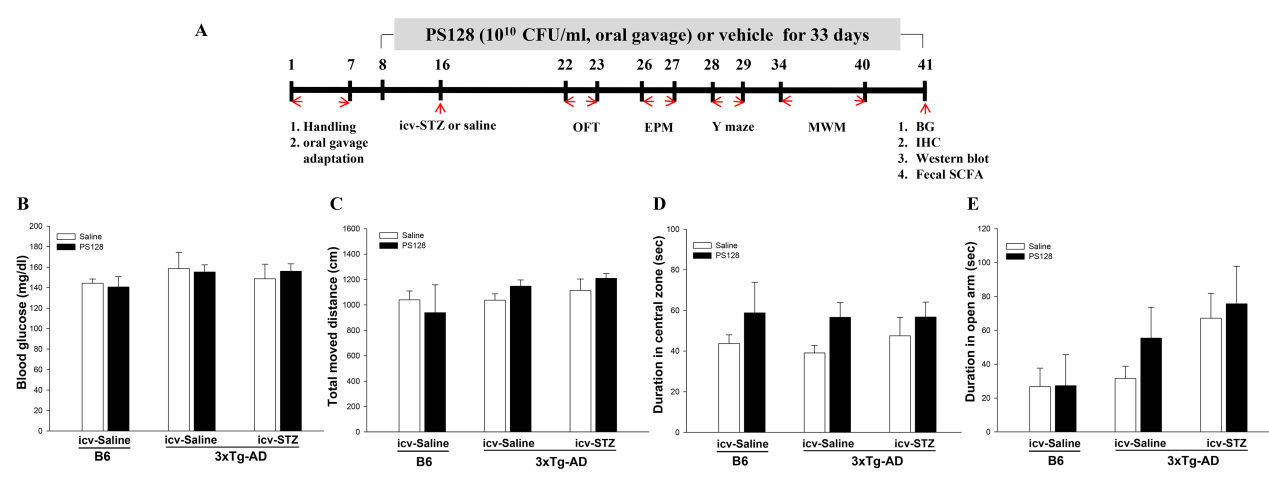


**Figure** **S1.** The effects of PS128 supplementation on anxiety behavior in 3×Tg-AD mice treated with icv-STZ. (A) The timeline of PS128 and icv-STZ treatment in mice. (B) Peripheral blood glucose levels in mice. The peripheral blood glucose levels were not different among all groups on day 47 (n = 15–20 animals /group). (C) The total distance traveled was not different among the groups, indicating the lack of a significant difference in the exploratory activity of all the groups (n = 15–20 mice/group). (D) The time spent in the central zone was not different among the mice in all groups (n = 15–20/group). (E) The time spent in the open arms was also not different among the mice in all groups (n = 15–20/group). These results indicated no difference in the anxiety behaviors of all the groups. The data presented as the means ± SEM. OFT, open field test; EPM, elevated plus maze; MWM, Morris water maze; BG, blood glucose; SCFA, short-chain fatty acid.


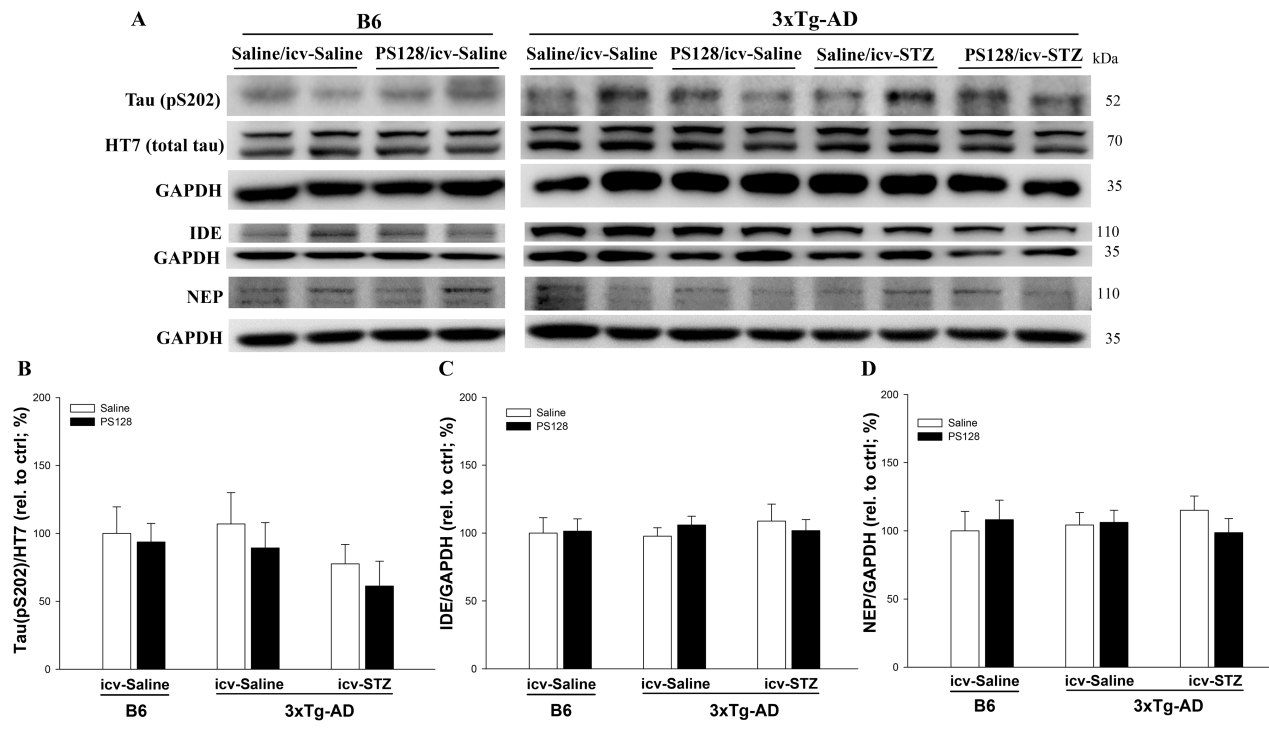


**Figure** **S2.** The effects of PS128 supplementation on the levels of the tau (pS202), IDE, and NEP protein expression in 3×Tg-AD mice treated with icv-STZ. (A) Representative image of western blots. (B-D) Quantitative densitometry results for the ratios of tau (pS202)/Ht7 (total tau), IDE/GAPDH, and NEP/GAPDH. GAPDH served as the internal control. The quantitative data are presented as the means ± SEM (n = 5–8 mice/group). IDE: insulin-degrading enzyme, NEP: neprilysin.

**
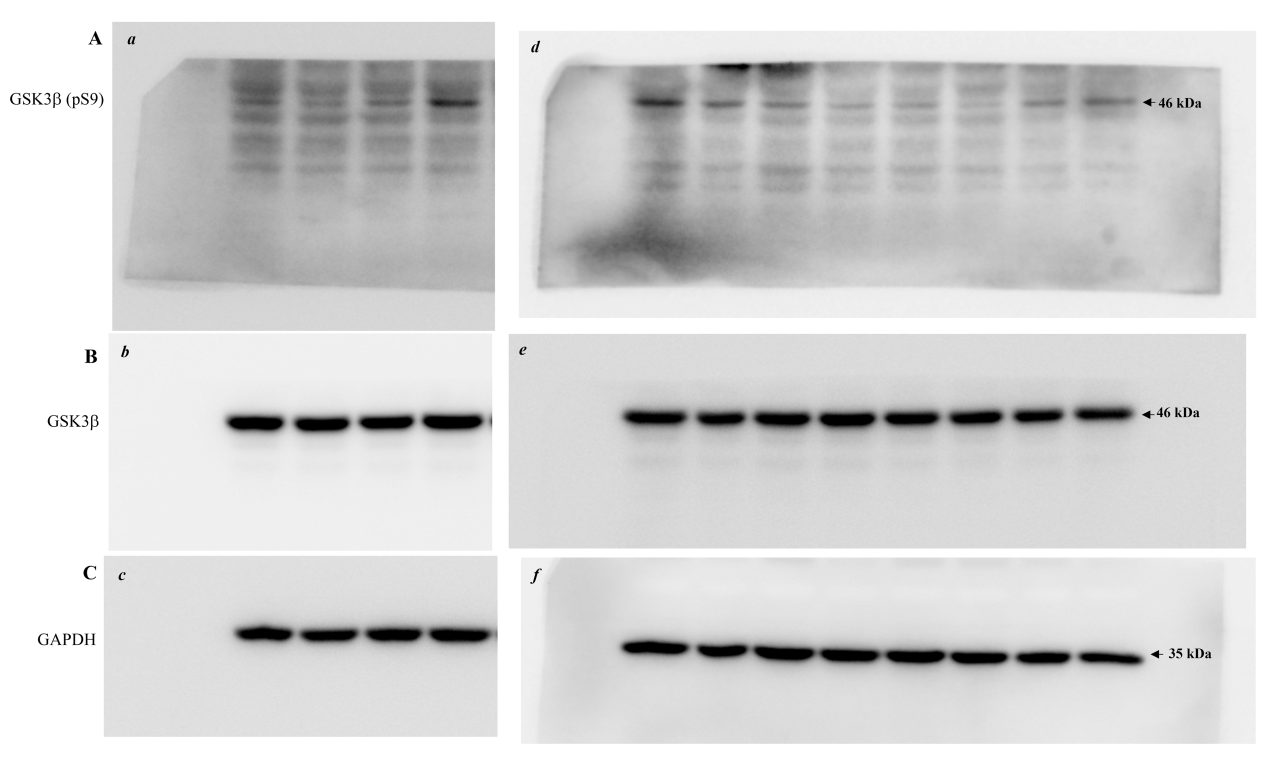
**

**Figure S3.** Original Uncropped Western blots for GSK3β related protein. (A) GSK3β (pS9), (B) GSK3β, and (C) GAPDH. (*a, b, c)* for B6 mice 1st-2nd lane: Saline/icv-Saline; 3rd-4th lane: PS128/icv-Saline. (*d, e, f)* for 3×Tg-AD mice 1st-2nd lane: Saline/icv-Saline; 3rd-4th lane: PS128/icv-Saline; 5th-6th lane: Saline/icv-STZ, 7th-8th lane: PS128/icv-STZ. 5th-6th lane: Saline/icv-STZ, 7th-8th lane: PS128/icv-STZ. Arrow indicated band site.

**
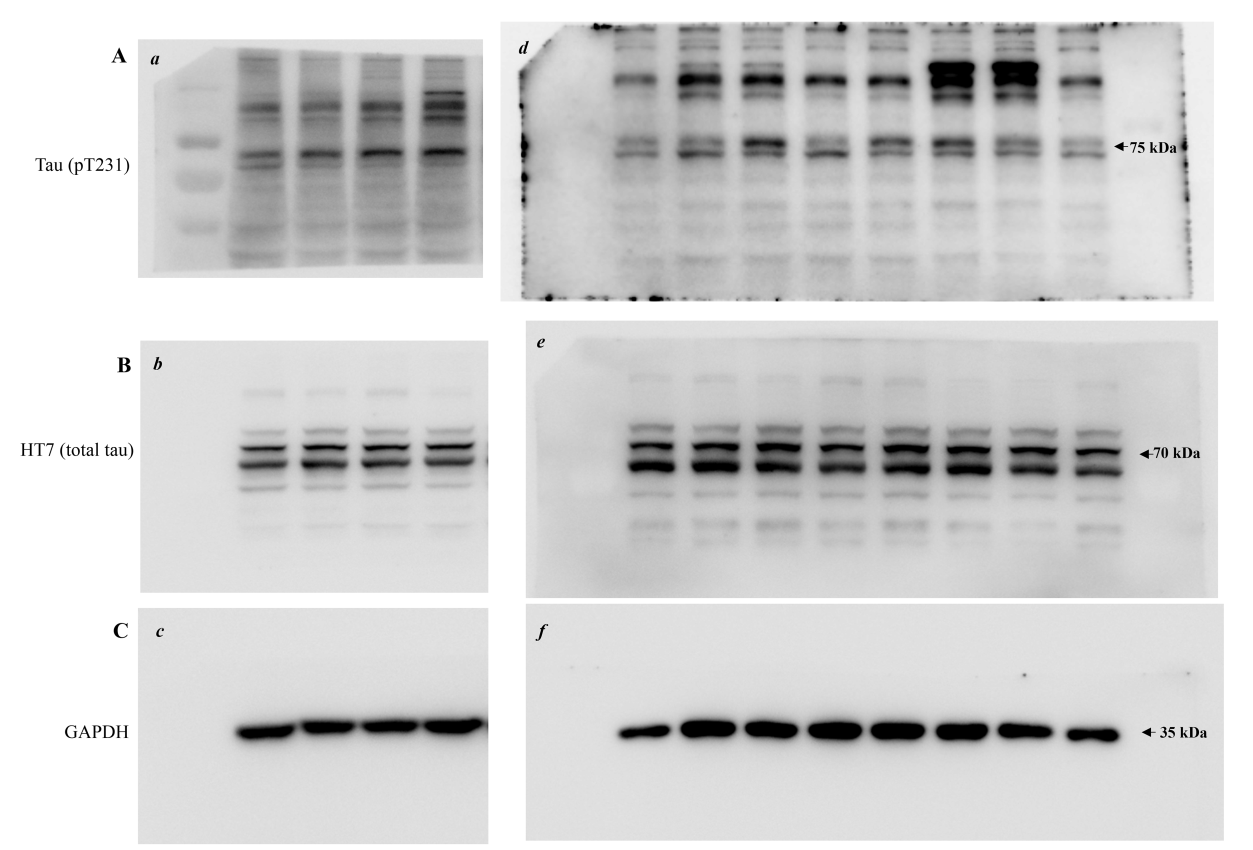
**

**Figure S4.** Original Uncropped Western blots for phosphorylated Tau protein. (A) pT231, (B) HT7 (total tau), and (C) GAPDH. (*a, b, c*) for B6 mice 1st-2nd lane: Saline/icv-Saline; 3rd-4th lane: PS128/icv-Saline. (*d, e, f*) for 3×Tg-AD mice 1st-2nd lane: Saline/icv-Saline; 3rd-4th lane: PS128/icv-Saline; 5th-6th lane: Saline/icv-STZ, 7th-8th lane: PS128/icv-STZ. 5th-6th lane: Saline/icv-STZ, 7th-8th lane: PS128/icv-STZ. Arrow indicated band site.


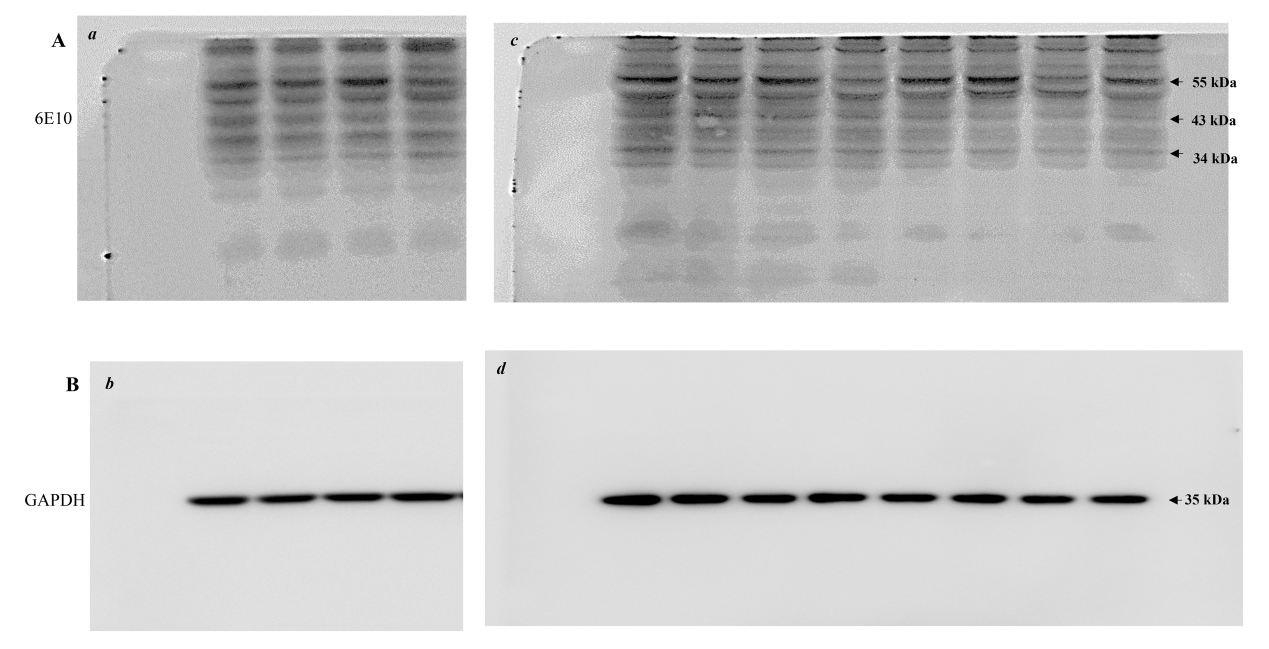


**Figure S5.** Original Uncropped Western blots for 6E10 protein. (A) 6E10, and (B) GAPDH. (*a, b*) for B6 mice 1st-2nd lane: Saline/icv-Saline; 3rd-4th lane: PS128/icv-Saline. (*c , d*) for 3×Tg-AD mice 1st-2nd lane: Saline/icv-Saline; 3rd-4th lane: PS128/icv-Saline; 5th-6th lane: Saline/icv-STZ, 7th-8th lane: PS128/icv-STZ. 5th-6th lane: Saline/icv-STZ, 7th-8th lane: PS128/icv-STZ. Arrow indicated band site.


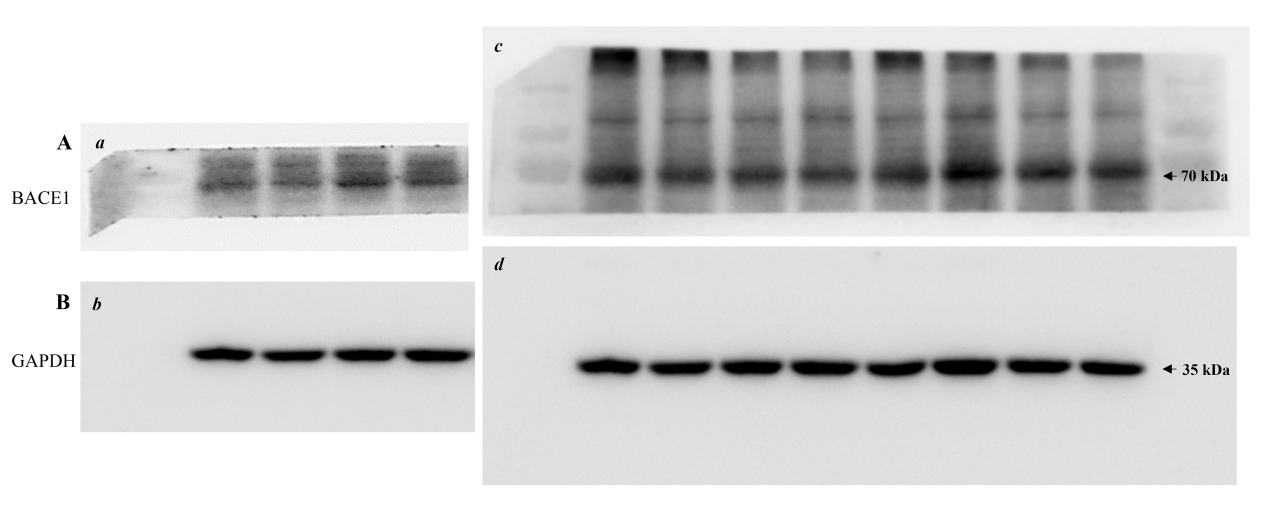


**Figure S6.** Original Uncropped Western blots for BACE1 protein. (A) BACE1, and (B) GAPDH. (*a, b*) for B6 mice 1st-2nd lane: Saline/icv-Saline; 3rd-4th lane: PS128/icv-Saline. (*c , d*) for 3×Tg-AD mice 1st-2nd lane: Saline/icv-Saline; 3rd-4th lane: PS128/icv-Saline; 5th-6th lane: Saline/icv-STZ, 7th-8th lane: PS128/icv-STZ. 5th-6th lane: Saline/icv-STZ, 7th-8th lane: PS128/icv-STZ. Arrow indicated band site.


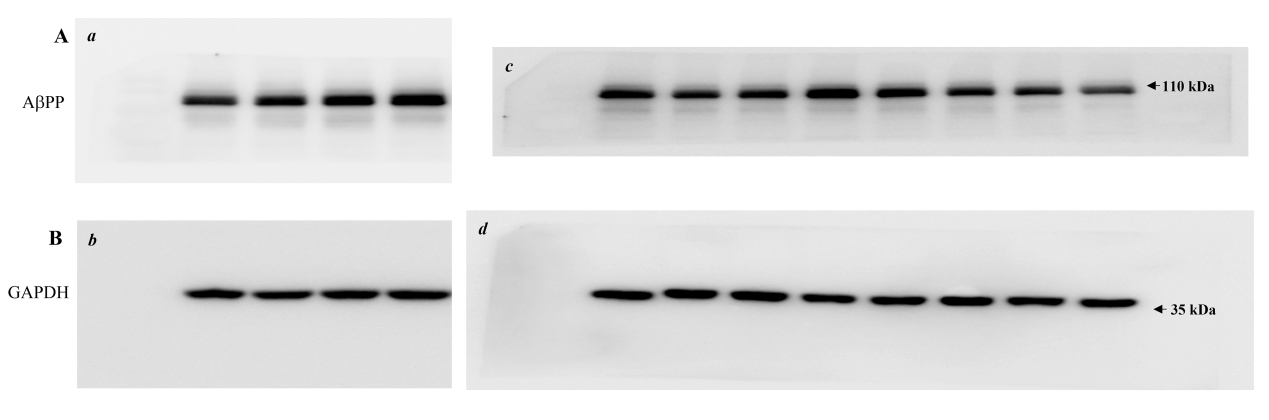


**Figure S7.** Original Uncropped Western blots for AβPP protein. (A) AβPP, and (B) GAPDH. (*a, b*) for B6 mice 1st-2nd lane: Saline/icv-Saline; 3rd-4th lane: PS128/icv-Saline. (*c, d*) for 3×Tg-AD mice 1st-2nd lane: Saline/icv-Saline; 3rd-4th lane: PS128/icv-Saline; 5th-6th lane: Saline/icv-STZ, 7th-8th lane: PS128/icv-STZ. 5th-6th lane: Saline/icv-STZ, 7th-8th lane: PS128/icv-STZ. Arrow indicated band site.


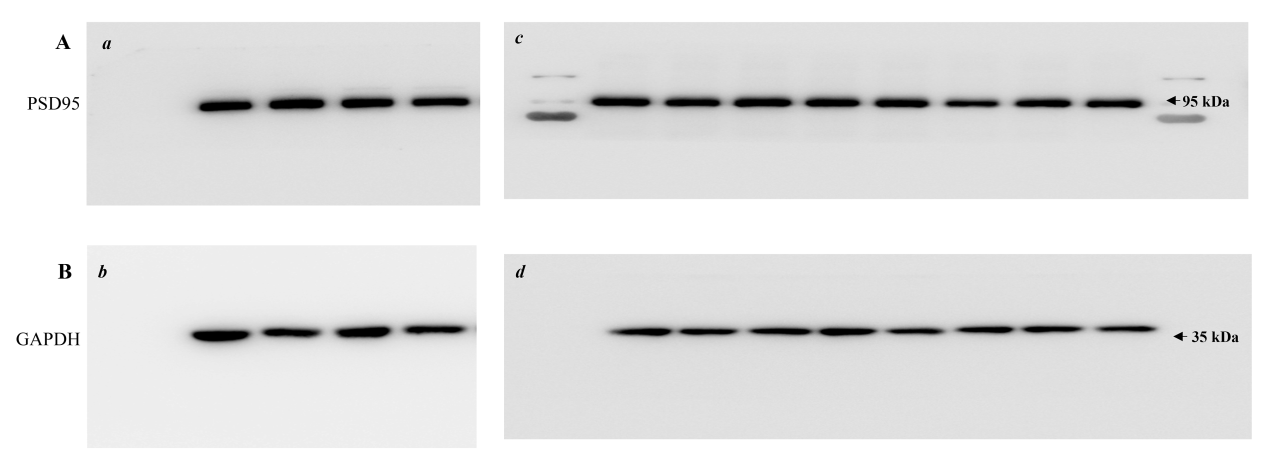


**Figure S8.** Original Uncropped Western blots for PSD95 protein. (A) PSD95, and (B) GAPDH. (*a, b*) for B6 mice 1st-2nd lane: Saline/icv-Saline; 3rd-4th lane: PS128/icv-Saline. (*c, d*) for 3×Tg-AD mice 1st-2nd lane: Saline/icv-Saline; 3rd-4th lane: PS128/icv-Saline; 5th-6th lane: Saline/icv-STZ, 7th-8th lane: PS128/icv-STZ. 5th-6th lane: Saline/icv-STZ, 7th-8th lane: PS128/icv-STZ. Arrow indicated band site.


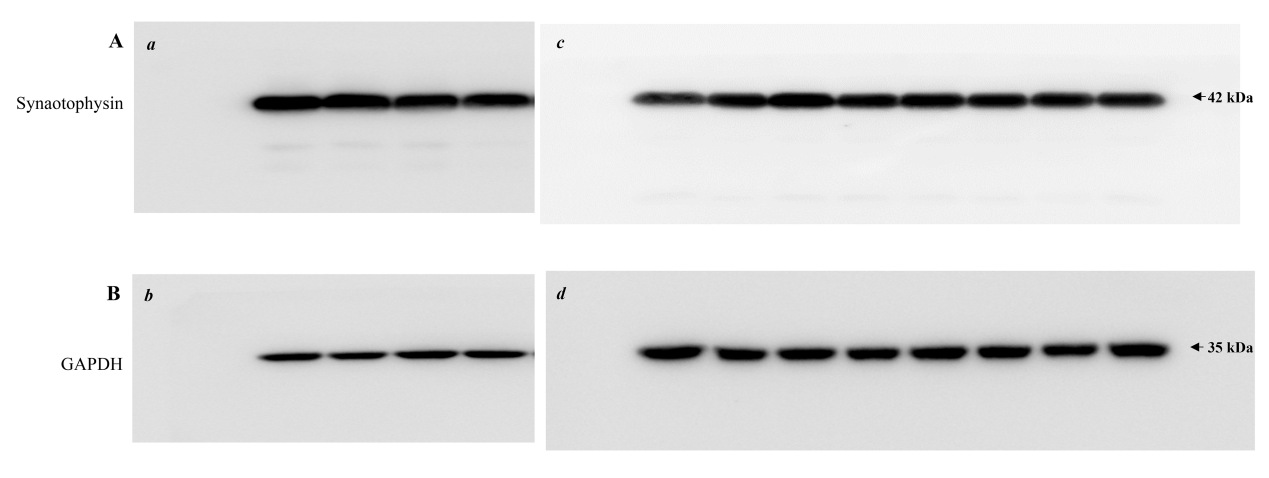


**Figure S9.** Original Uncropped Western blots for synaptophysin protein. (A) Synaptophysin, and (B) GAPDH. (*a, b*) for B6 mice 1st-2nd lane: Saline/icv-Saline; 3rd-4th lane: PS128/icv-Saline. (c, *d*) for 3×Tg-AD mice 1st-2nd lane: Saline/icv-Saline; 3rd-4th lane: PS128/icv-Saline; 5th-6th lane: Saline/icv-STZ, 7th-8th lane: PS128/icv-STZ. 5th-6th lane: Saline/icv-STZ, 7th-8th lane: PS128/icv-STZ. Arrow indicated band site.
